# Constitutional pericentric inversion of chromosome 16, inv(16)(p13.1q22), mimicking acute myeloid leukemia

**DOI:** 10.1002/jha2.603

**Published:** 2022-10-20

**Authors:** Shuji Ozaki, Honami Umeda, Makiko Mizuguchi, Yasunobu Okamoto, Hikaru Yagi, Kumiko Kagawa, Hironobu Shibata

**Affiliations:** ^1^ Department of Hematology Tokushima Prefecture Central Hospital Tokushima Japan; ^2^ Center for Medical Education Tokushima Prefecture Central Hospital Tokushima Japan

**Keywords:** constitutional chromosome rearrangement, inv(16)(p13.1q22), pericentric inversion

1

An acquired abnormality, inv(16)(p13q22), is a hallmark of acute myeloid leukemia with eosinophilia (AML M4Eo), which leads to leukemogenesis by forming the *CBFB::MYH11* fusion gene [1]. We experienced a case of inv(16)(p13.1q22) discovered incidentally during bone marrow examination without evidence of AML.

A 64‐year‐old woman was referred to our hospital because of hypergammaglobulinemia. She was being treated for rheumatoid arthritis, but had no past and family history of AML. Laboratory examination showed polyclonal gammopathy but no significant abnormalities in complete blood count with differential. Bone marrow picture showed a normocellular marrow and mild increase in plasma cells to 3.8% (reference range, 0.2%–1.7%), but no evidence of hematological malignancy. Surprisingly, chromosome analysis of the bone marrow cells revealed that all 20 cells had inv(16)(p13.1q22) mimicking AML M4Eo (Figure 1A). We therefore performed fluorescence in situ hybridization (FISH) analysis of the bone marrow cells by using the Vysis CBFB Dual Color Break Apart Rearrangement Probe (Abbott Molecular Diagnostics, Des Plaines, IL, USA). The splitting of the red and green signals reflecting truncation of the *CBFB* locus on chromosome 16q22 was not observed (Figure 1B). In addition, a multiplex reverse transcriptase‐polymerase chain reaction assay covering 28 leukemia‐specific fusion transcripts including *CBFB::MYH11* was performed by using the HemaVision‐28Q kit (VERITAS, Tokyo, Japan), but none of them was detected. Therefore, it was suggested that the chromosome inversion of this patient did not disrupt the *CBFB* gene. High‐resolution chromosome analysis of the phytohemagglutinin (PHA)‐stimulated peripheral blood lymphocytes also revealed inv(16)(p13.1q22) in all 20 cells, indicating a constitutional abnormality of chromosome 16 (Figure 1C). Thus, this patient was not misdiagnosed as AML M4Eo and has been followed up without treatment.

**FIGURE 1:**
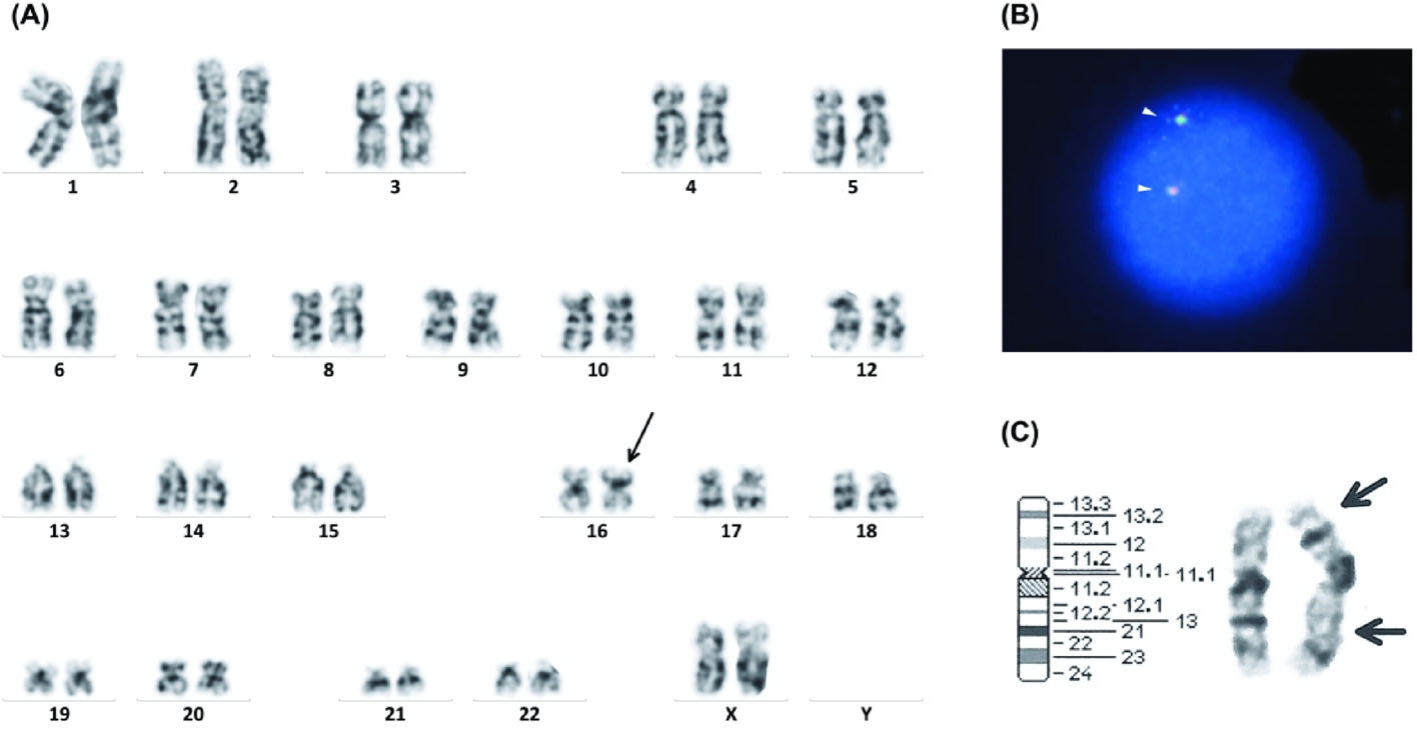
(A) Chromosome analysis of the bone marrow cells showing 46, XX, inv(16)(p13.1q22). An arrow indicates pericentric inversion of chromosome 16. (B) Fluorescence in situ hybridization (FISH) analysis of the bone marrow cells. The SpectrumRed probe hybridizes centromeric to the 16q22 breakpoint region, and the SpectrumGreen probe hybridizes telomeric to the breakpoint. Representative interphase cells showing two fused red/green (yellow) signals (arrowheads) showing no truncation of the *CBFB* locus on chromosome 16q22. (C) High‐resolution chromosome analysis of the phytohemagglutinin (PHA)‐stimulated peripheral blood lymphocytes showing inv(16)(p13.1q22). Arrows indicate the breakpoints of pericentric inversion of chromosome 16.

To the best of our knowledge, only a few cases with inv(16)(p13q22) unrelated to AML have been reported [2, 3, 4, 5]. All of these were concluded to be constitutional abnormalities, and some cases have been confirmed as familial onset. In these reports, there was no misdiagnosis of AML M4Eo in individuals without hematological malignancy, and in the case of chronic myeloid leukemia with t(9;22)(q34;q11) and inv(16)(p13q22), FISH analysis showed no abnormality of the *MYH11* gene and inv(16)(p13q22) was also found in her healthy father, which avoided misdiagnosis as an additional cytogenetic abnormality in chronic phase [4]. Therefore, although extremely rare, we should be aware of the presence of constitutional chromosome abnormalities mimicking hematological malignancies, and it is important to perform further tests such as FISH and genomic analysis for differential diagnosis.

## CONFLICT OF INTEREST

The authors declare that there is no conflict of interest.

## ETHICS STATEMENT

All procedures in this study were performed in accordance with the principles of the Declaration of Helsinki and the institutional guidelines.

## PATIENT CONSENT STATEMENT

Informed consent was obtained from the patient and her family.

## Data Availability

The data that support the findings of this study are available from the corresponding author upon reasonable request.
